# Influenza activity in Kenya, 2007–2013: timing, association with climatic factors, and implications for vaccination campaigns

**DOI:** 10.1111/irv.12393

**Published:** 2016-05-27

**Authors:** Gideon O. Emukule, Joshua A. Mott, Peter Spreeuwenberg, Cecile Viboud, Alexander Commanday, Philip Muthoka, Patrick K. Munywoki, David J. Nokes, Koos van der Velden, John W. Paget

**Affiliations:** ^1^ Centers for Disease Control and Prevention – Kenya Country OfficeNairobiKenya; ^2^ Department of Primary and Community CareRadboud University Medical CenterNijmegenThe Netherlands; ^3^ Influenza DivisionNational Center for Immunization and Respiratory DiseasesUS Centers for Disease Control and PreventionAtlantaGAUSA; ^4^ US Public Health ServiceRockvilleMDUSA; ^5^ Netherlands Institute for Health Services research (NIVEL)UtrechtThe Netherlands; ^6^ Fogarty International CenterNational Institutes of HealthBethesdaMDUSA; ^7^ Ministry of HealthGovernment of KenyaNairobiKenya; ^8^ Kenya Medical Research InstituteCentre for Geographic Medicine Research‐CoastKilifiKenya; ^9^ School of Life SciencesUniversity of WarwickCoventryUK

**Keywords:** Humidity, influenza, Kenya, respiratory, seasonality, vaccination

## Abstract

**Background:**

Information on the timing of influenza circulation remains scarce in Tropical regions of Africa.

**Objectives:**

We assessed the relationship between influenza activity and several meteorological factors (temperature, specific humidity, precipitation) and characterized the timing of influenza circulation and its implications to vaccination strategies in Kenya.

**Methods:**

We analyzed virologically confirmed influenza data for outpatient influenza‐like illness (ILI), hospitalized for severe acute respiratory infections (SARI), and cases of severe pneumonia over the period 2007–2013. Using logistic and negative binomial regression methods, we assessed the independent association between climatic variables (lagged up to 4 weeks) and influenza activity.

**Results:**

There were multiple influenza epidemics occurring each year and lasting a median duration of 2–4 months. On average, there were two epidemics occurring each year in most of the regions in Kenya, with the first epidemic occurring between the months of February and March and the second one between July and November. Specific humidity was independently and negatively associated with influenza activity. Combinations of low temperature (<18°C) and low specific humidity (<11 g/kg) were significantly associated with increased influenza activity.

**Conclusions:**

Our study broadens understanding of the relationships between seasonal influenza activity and meteorological factors in the Kenyan context. While rainfall is frequently thought to be associated with influenza circulation in the tropics, the present findings suggest low humidity is more important in Kenya. If annual vaccination were a component of a vaccination strategy in Kenya, the months of April to June are proposed as optimal for associated campaigns.

## Introduction

Influenza exerts a significant health burden on human populations across temperate, subtropical, and tropical regions.^1^, ^2^ In temperate regions, influenza epidemics exhibit clear seasonality with peaks during winter months^3^, ^4^ suggestive of an association with climatic factors. In these regions, lower temperature and lower specific humidity have been shown to be significantly associated with increased influenza activity.^5^, ^6^ In contrast, influenza seasonal characteristics are less predictable in tropical and subtropical regions which are characterized by semiannual epidemics or year‐round influenza activity.^5^, ^7^, ^8^, ^9^, ^10^ A meteorological factor that is frequently reported to be associated with high influenza incidences in the tropical areas is rainfall.^8^, ^9^, ^11^

In temperate countries, a well‐defined seasonality allows for a precise timing of influenza vaccination campaigns to precede periods of peak circulation. However, in tropical African countries, more data are needed on influenza seasonality and its determinants. In Kenya, where there is currently no influenza vaccination strategy in place, these data may help to inform vaccine implementation strategy decisions. Kenya experiences long rains that occur from March to May and short rains occurring in October and November. Temperatures are highest during the months of January to March.^12^ However, there is considerable climate variability within Kenya such that influenza surveillance has been set up in different locations, including the coastal tropical regions characterized by hot and humid weather year round; semiarid and desert‐like conditions in the northern and north eastern part of Kenya; and cooler highland locations in central and parts of western of Kenya. Data collected from influenza sentinel surveillance sites across the country have suggested increased influenza activity during rainy seasons,^11^, ^13^ but the full extent of how meteorological factors influence influenza activity is yet to be elucidated.

We assessed the relationship between the onset week of influenza activity as well as the weekly number of influenza cases with temperature, rainfall, and specific humidity during the years 2007–2013. We also described the patterns of periods of increased influenza circulation in different regions in Kenya and suggested possible implications for future vaccination programs.

## Methods

### Study sites and population

**Figure 1:**
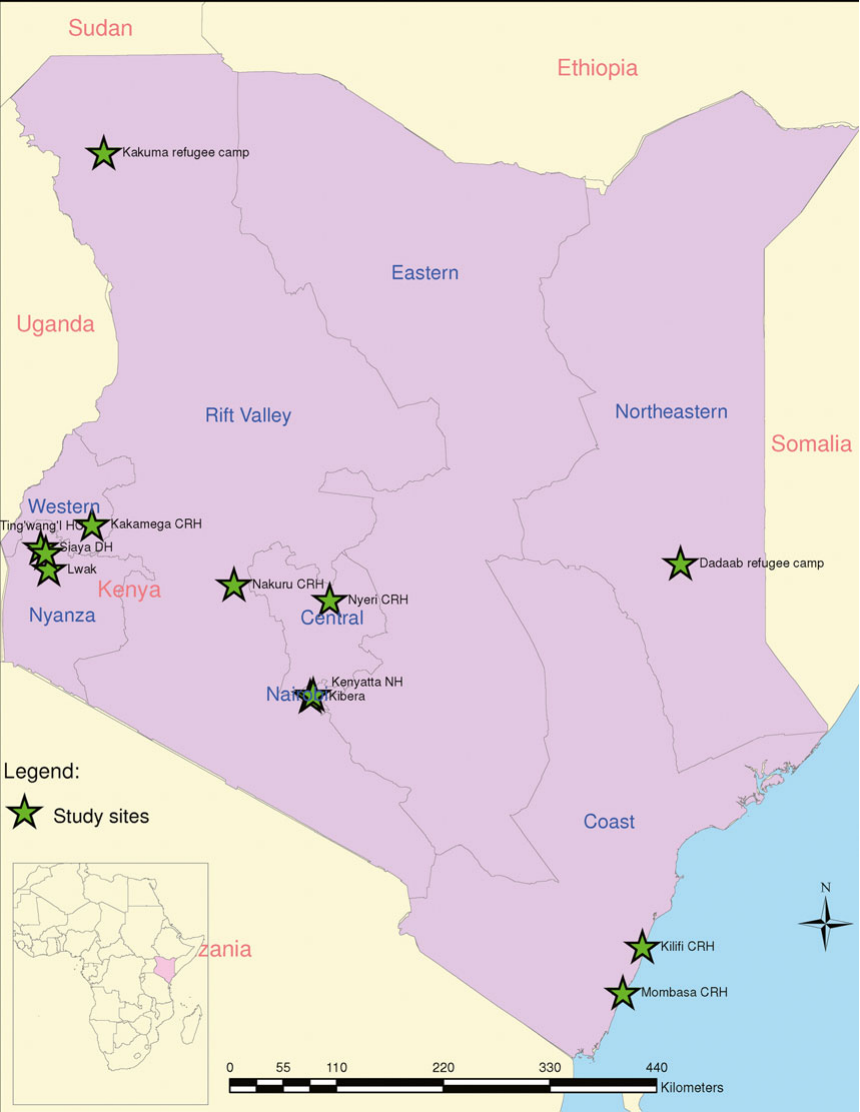
Map of Kenya showing the influenza surveillance sites.

We analyzed data collected between January 2007 and December 2013 from patients of all ages at all the 12 sites that conduct surveillance for influenza in Kenya. Included in our analysis were four sites from the western Kenya region; four sites from the central Kenya region; two sites from the northern/northeastern Kenya region; and two sites from the coastal Kenya region (Figure 1 and Table 1). These surveillance sites are representative of four climatic regions (western, central, northern/northeastern, and coastal) in Kenya.

**Table 1:** Descriptive statistics for influenza testing, January 2007–December 2013

Hospital/clinic	Period of data included in analysis	Number tested	Tested positive for influenza, *n* (%)	Hospitalized SARI cases tested	SARI cases tested positive for influenza, *n* (%)	Outpatient ILI/ALRI^a^ cases tested	ILI/ALRI cases tested positive for influenza, *n* (%)	Male, *n* (%)	Median age in years (IQR)
St. Elizabeth Hospital (Lwak)^b^	2007–2013	7493	1107 (14·8)	1162	105 (9·0)	6331	1002 (15·8)	3655 (48·8)	5·0 (2·1–12·8)
Siaya County Referral Hospital (CRH)	2010–2013	4769	325 (6·8)	4769	325 (6·8)	N/A	N/A	2373 (49·8)	1·8 (0·8–7·6)
Kakamega CRH	2008–2013	5801	630 (10·9)	3970	331 (8·3)	1831	299 (16·3)	3226 (55·6)	1·7 (0·8–3·5)
Ting'wang'i Health Center	2010–2013	1453	191 (13·1)	N/A	N/A	1453	191 (13·1)	690 (47·5)	2·2 (1·1–4·2)
**Western Kenya region**	**2007–2013**	**19 516**	**2253 (11·5)**	**9901**	**761 (7·7)**	**9615**	**1492 (15·5)**	**9944 (51·0)**	**2·7 (1·0–7·0)**
Kenyatta National Hospital	2008–2013	3576	268 (7·5)	2288	124 (5·4)	1288	144 (11·2)	2019 (56·5)	0·8 (0·5–1·5)
Tabitha Clinic (Kibera)^b^	2008–2013	6964	1263 (18·1)	N/A	N/A	6964	1263 (18·1)	3306 (47·5)	5·2 (1·9–15·3)
Nyeri CRH	2008–2013	4927	653 (13·3)	3159	351 (11·1)	1768	302 (17·1)	2741 (55·6)	1·3 (0·8–3·0)
Nakuru CRH	2008–2013	4138	561 (13·6)	2288	250 (10·9)	1850	311 (16·8)	2258 (54·6)	1·0 (0·7–2·2)
**Central Kenya region**	**2008–2013**	**19 605**	**2745 (14·0)**	**7735**	**725 (9·4)**	**11 870**	**2020 (17·0)**	**10 324 (52·7)**	**1·6 (0·8–4·4)**
Dadaab refugee camp	2008–2013	3064	571 (18·6)	2165	384 (17·7)	899	187 (20·8)	1713 (55·9)	1·4 (0·8–4·0)
Kakuma refugee camp	2007–2013	4942	649 (13·1)	3584	441 (12·3)	1358	208 (15·3)	2701 (54·7)	1·0 (0·7–3·0)
**Northern/Northeastern region**	**2007–2013**	**8006**	**1220 (15·2)**	**5749**	**825 (14·4)**	**2257**	**395 (17·5)**	**4414 (55·1)**	**1·0 (0·7–3·0)**
Mombasa CRH	2008–2013	2907	278 (9·6)	2097	171 (8·2)	810	107 (13·2)	1669 (57·4)	1·0 (0·5–2·0)
Kilifi CH^c^	2007–2013	5158	225 (4·4)	5158	225 (4·4)	N/A	N/A	2995 (58·1)	0·7 (0·2–1·5)
**Coastal Kenya region**	**2007–2013**	**8065**	**503 (6·2)**	**7255**	**396 (5·5)**	**810**	**107 (13·2)**	**4664 (57·8)**	**0·8 (0·3–1·7)**
**All sites**	**2007–2013**	**55 192**	**6721 (12·2)**	**30 640**	**2707 (8·8)**	**24 552**	**4014 (16·3)**	**29 346 (53·2)**	**1·7 (0·8–4·2)**

N/A; not applicable.

a Acute lower respiratory illness.

b Population‐based disease surveillance sites.

c At Kilifi CH, samples were collected from children <5 years who were hospitalized with severe or very severe pneumonia.

The western region receives more rainfall (1250–1700 mm annually) with average monthly temperatures ranging from 18 to 26°C. The central region has a relatively higher altitude compared to the other regions and experiences some of the lowest temperatures in the country (as low as 7°C during the July–August cold season). The northern/northeastern regions have semiarid and desert‐like conditions and experience sunny and dry weather most of the year. The coastal region experiences hot and humid weather conditions year round with average monthly temperatures ranging between 25 and 29°C and an average annual rainfall of over 1000 mm.^14^, ^15^

### Laboratory confirmation of influenza

Samples were collected from influenza‐like illness (ILI) outpatient case patients and hospitalized severe acute respiratory infection (SARI) case patients (Table 1).^13^ At Kilifi County Hospital (KCH), samples were collected from children <5 years who were hospitalized with severe or very severe pneumonia. The case definitions used are provided in Appendix S1. Samples collected were tested by real‐time reverse transcription polymerase chain reaction (rRT‐PCR) for influenza A and B viruses. Influenza A‐positive specimens were subtyped for A(H1N1), A(H3N2), and A(H1N1)pdm09.

### Influenza activity patterns

To assess influenza circulation over time, we calculated the average monthly proportion of influenza‐positive cases among those tested across the study period. Data from the pandemic period (August 2009–July 2010) were excluded because (i) these were likely to influence the pattern of results and (ii) we were interested in seasonal influenza. We also calculated the proportion of specimens that tested positive for influenza in the first half of the year within which the first epidemic typically occurs (January–June), and in the second half of the year within which the second epidemic occurs (July–December). Data from all the 12 surveillance sites were included in this analysis.

### Defining “influenza circulation periods”

“Influenza circulation periods” were defined as a period of ≥2 successive weeks where ≥10% of the total weekly cases tested were positive for influenza.^9^, ^16^ The 10% threshold was close to the average proportion (12%) of cases that tested positive for influenza over the entire study period. In situations where there were <25 cases tested in a week, we considered the proportions as unstable and used the five‐point moving average method to estimate the number of influenza cases and the percentage positive for influenza.^17^ Influenza circulation periods separated by ≥5 successive weeks where there was low influenza activity (<10%) were considered as two distinct periods. The first week of influenza circulation period is herein referred also as the start‐week or onset week (Figure S1).

### Meteorological data

The environmental data used in this analysis were satellite‐derived measurements and were collected over the same period of time as the influenza data. These variables were average surface temperature (°C) and near‐surface specific humidity (g/kg) obtained from the Global Land Data Assimilation System (GLDAS),^18^ and accumulated rainfall (mm) obtained from the Tropical Rainfall Measuring Mission (TRMM)^19^ (Appendix S1).

### Data analyses

#### Descriptive analyses for influenza and meteorological factors

Influenza circulation was described using proportions of influenza A‐ and/or B‐positive cases. The age distribution and the influenza activity patterns were described using medians and ranges. The meteorological factors were described using means and standard deviations (SD).

#### Bivariate and multivariate analyses of influenza activity

Data from nine of the 12 sites were used when assessing the associations between influenza activity and meteorological variables. Three surveillances sites (Kenyatta National Hospital, Mombasa County Referral Hospital, and Ting'wang'i Health Center) were excluded from these analyses because of multiple missing data points in the time series (Appendix S1).

We applied two analytical approaches to determine the association between influenza activity and meteorological variables: (i) logistic regression to determine the association between the onset of influenza activity and meteorological variables and (ii) negative binomial regression to determine the association between the weekly number of influenza cases and meteorological variables. In the first approach, the binary outcome variable “start‐week” was coded as “1” if the week considered was the onset week of influenza activity or otherwise coded as “0.” In the second approach, the outcome variable was the weekly count of influenza‐positive cases identified at each site. Negative binomial regression was chosen over the over Poisson regression to account for overdispersion in the data. The variables that were considered as covariates in the models were site, year, and week of the year (week = 1, 2, 3,…, 52). With the exception of the site and year variables which were analyzed as a categorical variables, all the other variables were entered into the respective models as continuous variables.

We investigated associations of up to 4 lagged weeks on all meteorological variables to assess a possible delayed weather effect on influenza activity (Appendix S1).^7^, ^18^ We additionally assessed whether “cold–dry” and “humid–rainy” conditions – as suggested in a recent global seasonality study – were associated with influenza activity.^5^ We used combinations of temperature and specific humidity at thresholds of <18°C and <11 g/kg, respectively, to define “cold–dry” conditions and combinations of specific humidity and rainfall at thresholds of >14 g/kg and >150 mm, respectively, to define “humid–rainy” conditions. In addition to investigating the effect of the “cold–dry” and “humid–rainy” conditions on influenza activity, we also assessed the effect of the two‐way product interaction (included as continuous variables) between temperature and specific humidity, and between specific humidity and rainfall. The interactions were evaluated in the model alongside the main effects of temperature, specific humidity, and rainfall.

The multiple variable models for the logistic and negative binomial regression analyses were fitted by including the site variable as well as all the variables that were associated with influenza in the bivariate analysis at overall *P*‐value < 0·2. Statistical significance was considered if the *P*‐value was <0·05.

All data analyses were performed using Stata version 13.0 (StataCorp. 2013, Stata Statistical Software: Release 13. College Station, TX: StataCorp LP).

### Ethical considerations

The study protocols were approved by both the institutional review board (IRB) of the U.S. CDC (CDC‐3308, CDC‐4566) and the ethical review committee of the Kenya Medical Research Institute (KEMRI; SSC‐1801, SSC‐932, SSC‐ 1161, SSC‐1055, 1526, 1858). At Nakuru, Kakamega, and Nyeri County Referral Hospitals, the Kenya Ministry of Health (KMoH) issued a letter stating that sentinel surveillance for influenza should be considered part of routine public health surveillance and therefore did not require formal ethical review. Verbal consent at these sites was obtained from all patients before questionnaires were administered and specimens were collected. For children, verbal consent was obtained from guardians.

## Results

### Descriptive analyses

A total of 55 192 patients were tested for influenza at the 12 surveillance sites over the period 2007 to 2013 of which 6721(12%) tested positive for influenza. The proportion of patients who tested positive for influenza ranged from 4% in Kilifi to 19% among patients who were seen at Dadaab refugee camp. The median age of the patients who were tested for influenza was 1·7 years [interquartile range (IQR) = 0·8–4·2 years; Table 1].

The mean average weekly temperature was lowest in Nakuru (18·2°C) and highest at Dadaab refugee camp (30·7°C; Table 2). The mean average weekly specific humidity ranged from 11·1 to 15·1 g/kg with Nyeri recording the lowest measurements, while Kilifi recorded the highest. The “cold–dry” conditions defined earlier were observed in only at two sites in the central Kenya region (Nakuru and Kibera). However, the “humid–rainy” conditions were only experienced at the coastal site (KCH) and at only four different time points (weeks) over the course of the study period.

**Table 2:** Descriptive statistics for the meteorological variables used in the analysis, January 2007–December 2013

Hospital/clinic	Period of data included in analysis	Yearly number of influenza circulation periods	Temperature (°C)		Specific humidity (g/kg)		Accumulated rainfall (mm)	
			Mean (SD)	Median (IQR)	Mean (SD)	Median (IQR)	Mean (SD)	Median (IQR)
St. Elizabeth Hospital (Lwak)	2007–2013	2	21·4 (1·5)	21·2 (20·3–22·3)	13·0 (1·3)	13·3 (12·4–13·9)	32·8 (27·7)	26·2 (10·5–46·2)
Siaya County Referral Hospital (CRH)	2010–2013	2	21·2 (1·4)	20·9 (20·3–21·9)	13·0 (1·2)	13·1 (12·5–13·7)	34·1 (28·4)	26·4 (10·6–49·0)
Kakamega CRH	2008–2011	1	21·4 (1·6)	21·2 (20·3–22·5)	13·2 (1·3)	13·4 (12·5–14·0)	32·9 (28·6)	25·1 (11·4–43·4)
Ting'wang'i Health Center^a^	N/A	–	N/A		N/A		N/A	
**Western Kenya region**	**2007–2013**	**2**	**21·4 (1·5)**	**21·2 (20·3–22·3)**	**13·0 (1·2)**	**13·3 (12·4–13·9)**	**32·8 (27·7)**	**26·2 (10·5–46·2)**
Kenyatta National Hospital^a^	N/A	–	N/A		N/A		N/A	
Tabitha Clinic (Kibera)	2009–2013	2	19·8 (1·3)	19·7 (18·8–20·7)	11·5 (1·3)	11·6 (10·7–12·6)	15·0 (24·1)	4·5 (1·1–19·3)
Nyeri CRH	2009–2012	2	19·8 (1·1)	19·6 (19·0–20·6)	11·1 (1·4)	11·3 (10·3–12·1)	21·2 (28·0)	9·5 (3·0–31·1)
Nakuru CRH	2009–2013	2	18·2 (1·2)	17·9 (17·3–18·8)	11·1 (1·5)	11·6 (10·4–12·3)	23·9 (21·5)	17·5 (7·6–33·8)
**Central Kenya region**	**2009–2013**	**2**	**19·2 (1·2)**	**19·0 (18·4–20·0)**	**11·3 (1·3)**	**11·5 (10·6–12·1)**	**20·0 (22·1)**	**11·9 (5·2–28·5)**
Dadaab refugee camp	2008–2009	2	30·7 (1·8)	30·9 (29·4–32·1)	13·4 (1·6)	13·1 (12·2–14·5)	5·8 (14·5)	0·0 (0·0–2·0)
Kakuma refugee camp	2007–2012	2	30·1 (1·7)	30·1 (29·1–31·1)	11·7 (2·1)	11·8 (10·2–13·3)	6·4 (11·7)	0·0 (0·0–7·2)
**Northern/Northeastern region**	**2007–2012**	**2**	**30·0 (1·8)**	**30·1 (29·1–31·2)**	**12·1 (2·0)**	**12·2 (10·9–13·5)**	**6·6 (11·8)**	**0·4 (0·0–8·4)**
Mombasa CRH^a^	N/A	–	N/A		N/A		N/A	
Kilifi CH	2007–2013	1	27·6 (1·5)	27·5 (26·4–28·7)	15·1 (1·5)	15·3 (13·7–16·3)	12·5 (29·0)	4·3 (1·2–12·4)
**Coastal Kenya region**	**2007–2013**	**1**	**27·6 (1·5)**	**27·5 (26·4–28·7)**	**15·1 (1·5)**	**15·3 (13·7–16·3)**	**12·5 (29·0)**	**4·3 (1·2–12·4)**
**All sites**	**2007–2013**	**2**	**24·77 (4·5)**	**25·5 (20·5–28·9)**	**13·0 (2·1)**	**13·1 (11·6–14·3)**	**18·3 (26·1)**	**8·1 (1·8–25·5)**

N/A, not applicable.

a Data from these sites were excluded from the analysis of association of influenza activity and meteorological variables because of multiple missing data points in the time series.

### Influenza activity patterns

A total of 48 periods of increased influenza circulation were identified across the nine study sites. Nineteen of these episodes occurred within the first quarter of the year (median onset month was February), 16 episodes occurred in the second quarter (median onset month was July), and the remaining 13 occurred in the last quarter (median onset month was October). On average, most of the study sites experienced two episodes of increased influenza circulation annually which lasted for a median duration of 2–4 months.

**Figure 2:**
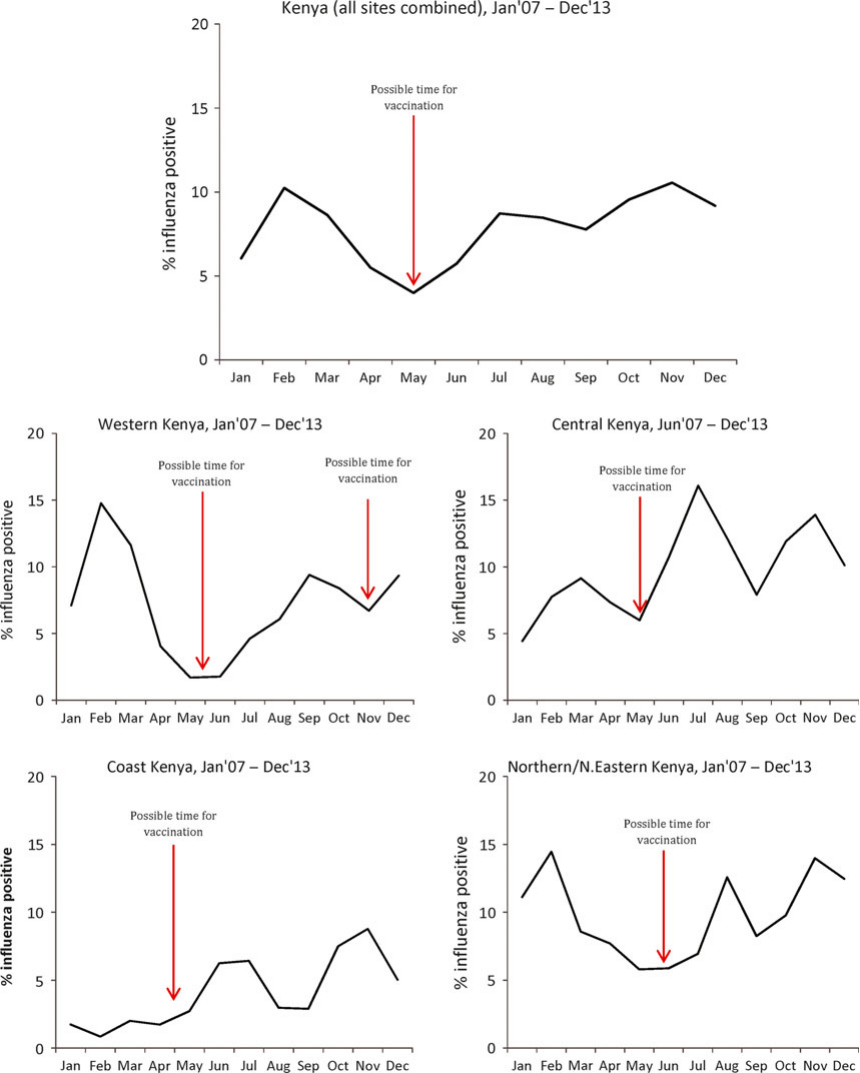
Monthly seasonal cycle of influenza activity in Kenya by region.

When we analyzed the monthly seasonal cycle of influenza activity, there was a pattern showing periods of increased influenza circulation occurring between February and March, and between July and November. There were more influenza‐positive cases identified in the last half of the years included in the analysis – within which the second epidemic occurs – compared to the first of half of the years [3886 (58%) influenza cases during July–December versus 2835 (42%) during January–June]. The month of May had the lowest influenza activity (Figure 2 and Figure S1).

### Bivariate and multivariable analyses

In the bivariate models for onset of influenza activity, specific humidity was significantly and negatively associated with the onset of influenza activity (*P* < 0·05). In the negative binomial regression models, influenza activity was found to be negatively associated with both temperature and specific humidity (*P* < 0·05). The presence of the “cold–dry” conditions, defined earlier, was also found to be significantly associated with influenza activity (*P* < 0·05). No statistically significant associations were observed between influenza activity and rainfall (Table 3).

**Table 3:** Bivariate analysis of the meteorological factors associated with influenza activity in Kenya, January 2007–December 2013

	Association with the onset of influenza activity		Absolute association with influenza activity	
	Odds Ratio (95% CI)	*P*‐value	Incidence Rate Ratio (95% CI)	*P*‐value
Year		0·233^a^		<0·001^a^
2007	0·70 (0·23–2·15)	0·538	0·45 (0·37–0·55)	<0·001
2008	0·74 (0·30–1·84)	0·516	0·78 (0·64–0·94)	0·010
2009	0·34 (0·10–1·18)	0·089	0·79 (0·62–1·00)	0·048
2010	0·31 (0·07–1·36)	0·120	0·96 (0·79–1·16)	0·649
2011	Ref		Ref	
2012	0·99 (0·46–2·11)	0·979	0·60 (0·49–0·74)	<0·001
2013	0·42 (0·14–1·27)	0·125	0·69 (0·56–0·85)	<0·001
Week	0·98 (0·97–1·00)	0·123	1·00 (0·99–1·00)	0·285
Site		0·813^a^		<0·001^a^
St. Elizabeth Hospital	Ref		Ref	
Tabitha Clinic (Kibera)	1·52 (0·56–4·12)	0·410	1·70 (1·36–2·11)	<0·001
Nyeri CRH	0·75 (0·19–2·85)	0·667	0·83 (0·64–1·08)	0·169
Kakamega CRH	1·00 (0·30–3·37)	1·000	1·07 (0·83–1·36)	0·609
Nakuru CRH	1·13 (0·39–3·30)	0·825	0·77 (0·61–0·97)	0·025
Siaya CRH	1·10 (0·35–3·41)	0·871	0·67 (0·53–0·85)	0·001
Dadaab refugee camp	1·44 (0·37–5·57)	0·594	0·94 (0·73–1·22)	0·649
Kakuma refugee camp	1·05 (0·38–2·94)	0·924	0·88 (0·71–1·09)	0·238
Kilifi County Referral Hospital	0·49 (0·15–1·66)	0·253	0·25 (0·19–0·33)	<0·001
Temperature (°C)				
No lag	0·99 (0·93–1·05)	0·682	0·97 (0·95–0·98)	<0·001
Lag 1 week	0·98 (0·92–1·04)	0·484	0·96 (0·95–0·98)	<0·001
Lag 2 weeks	0·97 (0·91–1·04)	0·350	0·96 (0·95–0·98)	<0·001
Lag 3 weeks	0·97 (0·91–1·04)	0·346	0·96 (0·95–0·98)	<0·001
Lag 4 weeks	0·97 (0·91–1·04)	0·394	0·96 (0·95–0·98)	<0·001
Specific humidity (g/kg)				
No lag	0·86 (0·75–0·98)	0·031	0·86 (0·84–0·89)	<0·001
Lag 1 week	0·82 (0·71–0·94)	0·004	0·85 (0·83–0·88)	<0·001
Lag 2 weeks	0·84 (0·73–0·96)	0·013	0·85 (0·82–0·87)	<0·001
Lag 3 weeks	0·89 (0·78–1·02)	0·108	0·84 (0·82–0·87)	<0·001
Lag 4 weeks	0·86 (0·75–0·99)	0·035	0·84 (0·82–0·86)	<0·001
Accumulated rainfall (mm)				
No lag	1·00 (0·99–1·01)	0·807	1·00 (1·00–1·00)	0·360
Lag 1 week	0·99 (0·98–1·00)	0·115	1·00 (1·00–1·00)	0·268
Lag 2 weeks	1·00 (1·00–1·01)	0·409	1·00 (1·00–1·00)	0·074
Lag 3 weeks	1·00 (0·99–1·01)	0·677	1·00 (1·00–1·00)	0·138
Lag 4 weeks	1·00 (0·99–1·01)	0·811	1·00 (1·00–1·00)	0·106
Presence of cold–dry conditions^b^				
No lag	8·17e‐06 (0–)	0·295	1·65 (1·09–2·50)	0·019
Lag 1 week	8·17e‐06 (0–)	0·295	2·05 (1·10–3·83)	0·024
Lag 2 weeks	8·17e‐06 (0–)	0·295	1·58 (0·98–2·53)	0·059
Lag 3 weeks	8·17e‐06 (0–)	0·295	2·12 (1·13–4·01)	0·020
Lag 4 weeks	8·18e‐06 (0–)	0·319	1·25 (0·74–2·11)	0·401

a Overall *P*‐value.

b Cold–dry periods were defined as weeks when the average temperature was <18°C and specific humidity was <11 g/kg.

In the multivariable logistic regression model, specific humidity was independently and negatively associated with the onset of influenza activity at lag weeks one [odds ratio (OR) = 0·79 (95% CI 0·66–0·94)] and two [OR = 0·82 (95% CI 0·69–0·98)] in the models that adjusted for the site variable. Similarly, specific humidity was significantly associated with influenza activity in the negative binomial regression models for the weekly count of influenza cases at the current week [incidence rate ratio (IRR) = 0·94 (95% CI 0·90–0·98)], and at all the four lag weeks investigated (*P* < 0·001). The presence of “cold–dry” conditions was also found to be positively associated with influenza activity when we adjusted for the site variable at current week [IRR = 1·90 (95% CI 1·20–3·01)], and at lag weeks one [IRR = 2·07 (95% CI 1·21–3·55)] and three [IRR = 1·95 (95% CI 1·11–3·44)]. However, temperature was not significantly associated with influenza activity when we adjusted for the site variable. All the other variables assessed including rainfall and the two‐way interactions between specific humidity and temperature and between specific humidity and rainfall were not significantly associated with influenza activity when we adjusted for the site variable (Table 4). An exploratory analysis to assess the relationship between the onset week of influenza activity and meteorological variables showed similar results to the ≥10% activity threshold when we used the median proportion (7% threshold) to define the onset of influenza activity (results not shown).

**Table 4:** Multivariable analysis of the meteorological factors associated with influenza activity in Kenya, January 2007–December 2013

	Association with the onset of influenza activity		Absolute association with influenza activity	
	Odds Ratio (95% CI)	*P*‐value	Incidence Rate Ratio (95% CI)	*P*‐value
Temperature (°C)				
No lag	1·06 (0·87–1·28)	0·573	1·03 (1·00–1·08)	0·086
Lag 1 week	0·97 (0·80–1·18)	0·752	1·01 (0·97–1·05)	0·720
Lag 2 weeks	0·90 (0·74–1·10)	0·308	1·01 (0·97–1·05)	0·750
Lag 3 weeks	0·90 (0·74–1·10)	0·299	0·99 (0·95–1·02)	0·464
Lag 4 weeks	0·93 (0·76–1·13)	0·443	0·98 (0·94–1·01)	0·219
Specific humidity (g/kg)				
No lag	0·85 (0·71–1·02)	0·078	0·94 (0·90–0·98)	0·005
Lag 1 week	0·79 (0·66–0·94)	0·007	0·91 (0·87–0·95)	<0·001
Lag 2 weeks	0·82 (0·69–0·98)	0·027	0·90 (0·86–0·94)	<0·001
Lag 3 weeks	0·91 (0·76–1·09)	0·307	0·88 (0·85–0·92)	<0·001
Lag 4 weeks	0·86 (0·71–1·02)	0·088	0·88 (0·85–0·91)	<0·001
Accumulated rainfall (mm)				
No lag	1·00 (0·99–1·01)	0·761	1·00 (1·00–1·00)	0·955
Lag 1 week	0·99 (0·97–1·00)	0·099	1·00 (1·00–1·00)	0·671
Lag 2 weeks	1·00 (0·99–1·01)	0·348	1·00 (1·00–1·00)	0·798
Lag 3 weeks	1·00 (0·98–1·01)	0·623	1·00 (1·00–1·00)	0·778
Lag 4 weeks	1·00 (0·99–1·01)	0·763	1·00 (1·00–1·00)	0·841
Presence of cold–dry conditions^a^				
No lag	NE	–	1·90 (1·20–3·01)	0·006
Lag 1 week	NE	–	2·07 (1·21–3·55)	0·008
Lag 2 weeks	NE	–	1·64 (0·97–2·78)	0·062
Lag 3 weeks	NE	–	1·95 (1·11–3·44)	0·021
Lag 4 weeks	NE	–	1·15 (0·59–2·24)	0·675

a Cold–dry periods were defined as weeks when the average temperature was <18°C and specific humidity was <11 g/kg.

NE, not estimated.

## Discussion

In this study, we found that there were multiple periods of increased influenza activity annually in Kenya. On average, there were two epidemics occurring each year in most of the regions in Kenya and these epidemics lasted a median duration of 2–4 months. The first epidemic occurred between February and March and the second between July and November. The period between April and May had the least influenza activity. We also identified that lower specific humidity was significantly associated with influenza activity in Kenya. As has been noted in other continents,^5^ we found that influenza was more likely to circulate when both temperature and specific humidity were below 18°C and 11 g/kg, respectively, independent of the study site. Contrary to what has been hypothesized previously,^5^ we found no significant association between influenza activity and rainfall.

Unlike temperate climates, the presence of multiple influenza epidemics each year in most of the regions in Kenya presents a challenge to the selection of the appropriate influenza vaccine formulation to use. Recent investigations have found that the Southern Hemisphere (SH) vaccine formulation was well matched [80% (95% CI 77–84) over a 9‐month period] to circulating strains over the period 2007–2013 (Waiboci *et al.,* accepted for publication in *Vaccine*). The Northern Hemisphere (NH) vaccine formulation was also well matched [82% (95% CI 78–85) over a 9‐month period]. These findings suggest that for the primary period of increased influenza circulation in Kenya (July–November), the SH vaccine formulation (available in April) could offer good protection. While this vaccine could also provide protection during the subsequent February and March peaks as well, the NH formulation (available in November) could also be considered for that period.

Our finding of a negative association between influenza activity and specific humidity is consistent with findings from other studies that were conducted in temperate^5^, ^20^ and subtropical regions. This is also consistent with experimental results which have linked low humidity to prolonged influenza virus survival (IVS) as well as efficient aerosol transmission.^21^, ^22^ These findings also suggest a site‐specific association between temperature and influenza activity as temperature was negatively and significantly associated with influenza activity in the bivariate analysis but not when we adjusted for the site variable. Whereas rainfall has previously been suggested to be correlated with influenza activity in tropical and subtropical regions,^5^, ^7^ our study did not find a significant association. A recent global study found that “humid–rainy” (high specific humidity and rainy) conditions were associated with influenza circulation.^5^ However, our analysis did not support this finding. The lack of association between the “humid–rainy” conditions with influenza activity in our study context may in part be explained by the fact that we do not experience necessary thresholds of high specific humidity (>14 g/kg) and high rainfall (>150 mm) measurements as previously suggested.^5^ Indeed, these conditions were only experienced at four different time points over the course of the study period at the coastal site (KCH).

The relative merits of annual influenza vaccination versus the integration of influenza vaccination into routine immunization schedules remain to be evaluated in Kenya and are beyond the scope of this discussion. However, if annual mass vaccination campaigns are being considered, the period between April and June would perhaps be the optimal time for several reasons. First, this would potentially offer better protection considering the fact that the period of influenza activity between July and December account for most of the annual influenza cases (58%). Considering the possibility of waning immunity over time,^23^, ^24^ it would probably be preferable to vaccinate during the month of June. However, a wider period may need to be considered in the context of the possible logistical challenges of vaccine delivery and accessing the target populations. Second, according to the Kenyan education calendar, schools are closed for holidays during the months of April, August, and December. April would therefore be a more convenient time for school‐going children to be immunized in non‐school settings. Lastly, caretakers of young children may take advantage of the presence of older children during these holidays to take care of household chores as they attend to other health‐related matters such as taking the smaller children for immunization.

Our study was subject to some other important limitations. First, we were not able to account for the effect of other factors such as socialeconomic conditions, population susceptibility, and human migration dynamics on the association between influenza activity and meteorological variables because these data were not collected. Second, we only relied on satellite‐derived meteorological measurements for our analysis. Even though we had a reasonable temporal resolution in the meteorological data, using actual ground data could possibly have provided more accurate results. Third, although we tried to adjust for the effect of the site differences in our models, we could not sufficiently explore the regional variation of meteorological factors in Kenya and how they correlate with influenza activity because of limited influenza testing data available. Lastly, we could not explore whether the association between influenza activity and meteorological factors varied by age as older persons were underrepresented in the hospital‐based surveillance because of low healthcare seeking behavior.^25^

In conclusion, our study broadens our understanding of the relationships between seasonal influenza activity and meteorological factors in tropical regions, and more specifically in the Kenyan context. We additionally highlight the influenza activity patterns in Kenya with regard to the onset months of periods of increased influenza circulation. These could help to inform the timing of future influenza vaccination campaigns in Kenya and highlight periods when added diagnostic measures, treatment efforts, or infection control strategies may be put in place.

## Author contributions

Gideon O. Emukule, Joshua A. Mott, Peter Spreeuwenberg, Cecile Viboud, D. James Nokes, Koos van der Velden, and John W. Paget involved in concept and design of manuscript; Gideon O. Emukule analyzed the data and lead author in writing the manuscript; Gideon O. Emukule, Joshua A. Mott, Peter Spreeuwenberg, Cecile Viboud, Philip Muthoka, Patrick K. Munywoki, D. James Nokes, Koos van der Velden, and John W. Paget involved in interpretation of data; Gideon O. Emukule, Joshua A. Mott, Peter Spreeuwenberg, Cecile Viboud, Alexander Commanday, Patrick K. Munywoki, D. James Nokes, Koos van der Velden, and John W. Paget helped in writing of the manuscript; and Alexander Commanday involved in literature review.

## Funding

This study was supported through The KEMRI and U.S. CDC research collaboration and The Wellcome Trust, UK. The funders had no role in study design, data collection and analysis, decision to publish, or preparation of the manuscript.

## Disclaimer

The findings and conclusions in this report are those of the authors and do not necessarily represent the official position of the Centers for Disease Control and Prevention.

## Supporting information


**Figure S1.** Figures showing the time series of the average weekly temperature, specific humidity and percent influenza positive cases by site, 2007 to 2013.

**Appendix S1.** Supplemental methods.
