# Supplementary material for: Influenza activity in Kenya, 2007–2013: timing, association with climatic factors, and implications for vaccination campaigns
Source: Influenza Other Respir Viruses. 2016 May 27;10(5):375–85. doi: 10.1111/irv.12393 (PMC4947939; doi:10.1111/irv.12393)
Supplement: Supplementary file 1 — Figure S1. Figures showing the time series of the average weekly temperature, specific humidity and percent influenza positive cases by site, 2007 to 2013. [file IRV-10-375-s001.docx]

**Figure S1.** Supplemental figures showing the time series of the average weekly temperature, specific humidity and percent influenza positive cases by site, 2007 to 2013

The vertical lines indicates the identified onset week and the dotted horizontal line shows the 10% infleunza positive cut-off point.

**Definitions:**

1. *Cold-dry* week was defined as the week when temperature was <18^0^C and specific humidity was <11g/kg.
2. *Humid-rainy* week was defined as the week when specific humidity was >14g/kg and rainfall was >150mm.
