# Supplementary material for: Influenza activity in Kenya, 2007–2013: timing, association with climatic factors, and implications for vaccination campaigns
Source: Influenza Other Respir Viruses. 2016 May 27;10(5):375–85. doi: 10.1111/irv.12393 (PMC4947939; doi:10.1111/irv.12393)
Supplement: Supplementary file 2 — Appendix S1. Supplemental methods. [file IRV-10-375-s002.docx]

**Appendix S1. Supplemental methods**

**Case definitions**

*Influenza-Like Illness*

Influenza-like illness was defined as an axillary temperature ≥38^o^C and cough or sore throat in an outpatient of any age [[1](#_ENREF_1), [2](#_ENREF_2)].

*Severe Acute Respiratory Illness (SARI)*

SARI was defined differently for children under five years and for persons ≥5 years. Among children aged <5 years, SARI was defined using a modified version of the World Health Organization’s Integrated Management of Childhood Illness (IMCI) definition for pneumonia. This was defined as hospitalization with cough OR difficulty breathing, AND at least one of (maternal report of lower-chest wall in-drawing, stridor in a calm child, unable to drink or breast feed, vomiting, convulsions, lethargic or unconscious, oxygen saturation <90%). Among persons aged 5 years, SARI was defined as hospitalization with cough OR difficulty breathing OR shortness of breath AND a documented fever (≥38^o^C) [[1](#_ENREF_1), [2](#_ENREF_2)].

At the Kilifi site, severe pneumonia was defined as cough OR difficult breathing AND lower chest wall in-drawing and no signs of very severe pneumonia. Very severe pneumonia was defined as cough OR difficult breathing AND at least one of (hypoxia (oxygen saturation <90%), cyanosis, prostration (inability to drink or breast feed, inability to sit), or coma at admission) [[3](#_ENREF_3)].

**Missing data**

We applied several procedures to handle missing data at each site. First, we excluded data for the whole year if there were five or more successive weeks with no influenza testing conducted. Second, if no data were collected in a specific week but data were collected in the previous and succeeding weeks, we substituted the missing data point by the average of two immediate weeks (one from each side). This applied to 2% of the weeks in the entire dataset. Lastly, for cases where data were missing for 2-4 successive weeks, we substituted the missing observation with data from the last week when influenza testing was conducted. This applied to 3% of the weekly data points in the dataset. Overall, we obtained 1,872 weeks of influenza surveillance data for further analyses of which 5% were imputed.

**Meteorological Data**

All the environmental data used in this analysis were satellite-derived measurements obtained from The National Aeronautics and Space Administration (NASA), and were collected over the same period as the influenza data. The environmental variables included in the analysis were, average surface temperature (^0^C), and near surface specific humidity (g/Kg) obtained from the Global Land Data Assimilation System (GLDAS) [[4](#_ENREF_4)]; and accumulated Rainfall (mm) obtained from the Tropical Rainfall Measuring Mission (TRMM) [[5](#_ENREF_5)]. The temperature and specific humidity measurements were all three-hourly temporal datasets with a spatial resolution of 1.0^0^ x 1.0^0^ latitude/longitude. The rainfall data were daily temporal datasets and had a finer resolution of 0.25^0^ x 0.25^0^ latitude/longitude. These data were downloaded through NASA’s Goddard Earth Sciences and Data Information Service Center (GES-DISC) Interactive Online Visualization and Analysis Infrastructure (GIOVANNI). All the meteorological measurements (3-hourly and daily) were then averaged over each week to obtain the weekly measurements which were used in the final analysis.

To determine if influenza was associated with earlier measurements of the meteorological variables, we investigated associations of up to 4 lagged weeks on all meteorological variables. We selected this lag period because the exploratory analyses indicated no association when we considered lag periods beyond four weeks. Also given that the influenza incubation period ranges from 1-4 days after exposure and that the infected persons could continue to be infectious for another 5-7 days after the onset of symptoms [[6](#_ENREF_6)] in normal circumstances, we believed that 4 weeks was an optimal period for an epidemic to start and spread into the community.

**References**

1. Katz MA, Lebo E, Emukule G, Njuguna HN, Aura B, Cosmas L, et al. Epidemiology, seasonality, and burden of influenza and influenza-like illness in urban and rural Kenya, 2007-2010. The Journal of infectious diseases. 2012;206 Suppl 1:S53-60. doi: 10.1093/infdis/jis530. PubMed PMID: 23169973.

2. Katz MA, Muthoka P, Emukule GO, Kalani R, Njuguna H, Waiboci LW, et al. Results from the first six years of national sentinel surveillance for influenza in Kenya, July 2007-June 2013. PloS one. 2014;9(6):e98615. doi: 10.1371/journal.pone.0098615. PubMed PMID: 24955962; PubMed Central PMCID: PMC4067481.

3. Onyango CO, Njeru R, Kazungu S, Achilla R, Bulimo W, Welch SR, et al. Influenza surveillance among children with pneumonia admitted to a district hospital in coastal Kenya, 2007-2010. The Journal of infectious diseases. 2012;206 Suppl 1:S61-7. doi: 10.1093/infdis/jis536. PubMed PMID: 23169974; PubMed Central PMCID: PMC3502370.

4. NASA Goddard Space Flight Center. Global Land Data Assimilation System. Available at: <http://gdata1.sci.gsfc.nasa.gov/daac-bin/G3/gui.cgi?instance_id=GLDAS10_M>. Date Accessed: April, 2014. 2014.

5. NASA Goddard Space Flight Center. Tropical Rainfall Measuring Mission. Available at: <http://gdata1.sci.gsfc.nasa.gov/daac-bin/G3/gui.cgi?instance_id=TRMM_Monthly>. Date Accessed: April, 2014. 2014.

6. Centers for Disease Control and Prevention. How Flu Spreads. Available at: <http://www.cdc.gov/flu/about/disease/spread.htm>. Accessed on October 4th, 2014. 2013.
